# Paracellular and Transcellular Leukocytes Diapedesis Are Divergent but Interconnected Evolutionary Events

**DOI:** 10.3390/genes12020254

**Published:** 2021-02-10

**Authors:** Michel-Edwar Mickael, Norwin Kubick, Pavel Klimovich, Patrick Henckell Flournoy, Irmina Bieńkowska, Mariusz Sacharczuk

**Affiliations:** 1 Department of Experimental Genomics, Institute of Animal Biotechnology and Genetics, Polish Academy of Science, Postępu 36A, 05-552 Jastrzebiec, Poland; keramina.poland@gmail.com; 2 Department of Immunology, PM Forskningscentreum, 17854 Ekerö Stockholm, Sweden; klimovichpavlusha@gmail.com (P.K.); pflournoy@philomako.org (P.H.F.); 3 Department of Biochemistry and Molecular Cell Biology (IBMZ), University Medical Center Hamburg-Eppendorf, Martinistraße 52, 20246 Hamburg, Germany; n.kubick@uke.de; 4 Department of Pharmacodynamics, Faculty of Pharmacy, Warsaw Medical University, l Banacha 1, 02-697 Warsaw, Poland

**Keywords:** paracellular, transcellular, diapedesis, PECAM1, CAV1

## Abstract

Infiltration of the endothelial layer of the blood-brain barrier by leukocytes plays a critical role in health and disease. When passing through the endothelial layer during the diapedesis process lymphocytes can either follow a paracellular route or a transcellular one. There is a debate whether these two processes constitute one mechanism, or they form two evolutionary distinct migration pathways. We used artificial intelligence, phylogenetic analysis, HH search, ancestor sequence reconstruction to investigate further this intriguing question. We found that the two systems share several ancient components, such as RhoA protein that plays a critical role in controlling actin movement in both mechanisms. However, some of the key components differ between these two transmigration processes. CAV1 genes emerged during Trichoplax adhaerens, and it was only reported in transcellular process. Paracellular process is dependent on PECAM1. PECAM1 emerged from FASL5 during Zebrafish divergence. Lastly, both systems employ late divergent genes such as ICAM1 and VECAM1. Taken together, our results suggest that these two systems constitute two different mechanical sensing mechanisms of immune cell infiltrations of the brain, yet these two systems are connected. We postulate that the mechanical properties of the cellular polarity is the main driving force determining the migration pathway. Our analysis indicates that both systems coevolved with immune cells, evolving to a higher level of complexity in association with the evolution of the immune system.

## 1. Introduction

Regulation of leukocytes infiltration through the endothelial cells of the BBB is an intriguing phenomenon that plays a vital role in homeostasis [1]. In homeostasis, high BBB integrity and lack of lymphocyte adhesion molecules reduce immune cells infiltration of the brain. BBB integrity is assured by two factors [2]. The first is the existence of tight junctions that are controlled by two complexes, the first complex consists of ZO1, ZO2, and ZO3 as well as occludin [3]. The second complex is composed of junctional adhesion molecules and there are three of them (JAM1, JAM2, and JAM3) in addition to ESAM [4]. The second factor is the existence of cadherins which represent the adhesion junctions between adjacent endothelial cells [5]. The main protein that controls this process is VE-Cadherin as it is connected to the cell’s actin through alpha and beta-catenin [6,7,8,9]. This compact system ensures that leukocytes’ access to the brain is limited during homeostasis.

During neuro-inflammatory pathologies the permeability of the endothelial cells changes to increase the probability of successful infiltrations of the lymphocytes to the brain through paracellular or transcellular migration routes [10,11,12,13]. In paracellular transmigration a series of signaling cascade takes place, including upregulation of lymphocytes adhesion molecules such as VCAM1 and ICAM1 as well as activation signals such as CC20 and CCL21. These interactions ensure capturing and adhesion of lymphocytes [14]. Once activated ICAM 1 and VCAM1 in turn activate the RhoA and Src pathway, that phosphorylates the β-catenin thus freeing VE-cadherin [15]. Following that, LBRC (lateral border recycling compartment) stores the junctional proteins [16,17]. PECAM1 expressed on both endothelial cells and migrating CD4+ T cells is upregulated to facilitates the motion of the CD4+ in the space between the cells through homophilic bindings [9]. Transcellular migration could constitute one third of the cases of diapedesis [18]. Interestingly, transcellular migration increases in neuro-inflammatory conditions such as multiple sclerosis [19]. One of the main differences between transcellular migration and paracellular migration is the presence membrane fusion process that employs vesicles enriched with caveolae marker (CAV1) as well as vesiculo-vacuolar organelle (VVO) in addition to SNARE proteins that play an important role in transcellular pore formation [20].

### The Origin and the Nature of These Systems Is Not Yet Known

The evolutionary origin of these two systems is not yet known. Whether they constitute the same physiological process is still debated. One side of the argument supports the notion that paracellular and transcellular diapedesis are physiologically identical with non-critical differences [16]. This point of view suggests that the occurrence of one system rather than the other is primarily based on leukocytes infiltration ability. In particular, the probability leucocytes probing the space between the endothelial cells or the endothelial cells proper is based on the resistance needed by the leucocytes to infiltrate that specific area. Conversely, it has been shown that CAV1 expression favors trans-cellular migration, and CAV1^−/−^ mice only exhibit paracellular migration [21]. Similarly, in PECAM1^−/−^ mice, leukocytes migration into the brain exhibited transcellular migration [9]. Thus, the question rises, if these two systems were identical, what would be the advantage of having two non-redundant systems. Intriguingly, in drosophila, a para-cellular migration system has been demonstrated in germ cells [22]. However, it is not yet known if a similar mechanism in the CNS. Thus, further investigative studies are needed to disentangle the evolutionary origins and pathways controlling these vital mechanisms.

In this research, we compared the evolution of paracellular and transcellular transmigration mechanisms using a phylogenetic approach. First, we used an AI text mining method to extract the gene names that are expressed in any of these two transmigration systems. We built multiple sequence alignments and phylogenetic trees for each of these components. Then, we identified genes that play a critical role in controlling the leukocytes transmigration route, namely, PECAM1 and CAV1. One of the critical differences between paracellular and transcellular migration pathways is the role of caveolae manifested by CAV1 which is as ancient as *Trichoplax adhaerens*. On the other hand, PECAM1 plays an indispensable role in paracellular diapedesis, and it appeared during the divergence of the zebrafish from FCRL5. Our results indicate that both systems contain ancient components such as RhoA which is more than one billion years old. Moreover, both systems share newly emergent components such as ICAM1 and VCAM1. Collectively, our results suggest that there are at least two different systems of endothelial migration in the brain. These systems are characterized by several unique components such as PECAM1 and CAV1. However, there is considerable interconnection between them.

## 2. Methods

### 2.1. Text Mining Using AI

The workflow for this step was as follows. First, we used Google Scholar to search for relevant papers (in PDF format) using the terms; transmigration, paracellular, transcellular, diapedesis, extravasation and “brain”. The number of papers downloaded and used to build a JSON format database was 30. The JSON format used ensured preserving a separate structure for the constituents of each PDF file.

The database was searched using our built-in AI search tool (AIST) [23]. The input to AIST was the question “What are the genes that affect endothelial migration?”, in addition to our database. This input was fed into a google auto-encoder to calculate the embedding (numerical matrix representation of the text) of each sentence. After that, we calculated the relevance between each sentence in the database, and the question posed using a convolutional 2D network as described earlier. The genes identified from each PDF were stored, and the gene names extracted. We used a cutoff of 99 genes to stop the search. After that, we manually grouped the genes into five categories (i) genes that play a role in paracellular transmigration (ii) genes that play a role in transcellular transmigration (iii) genes that play a role in both paracellular and transcellular migration (iv) genes that are expressed mainly by leukocytes (v) non-relevant genes. Only the first three categories were used in further analysis.

### 2.2. Data Used and Phylogenetic Tree

Next, we examined the evolutionary history of paracellular and transcellular transmigration pathways for the genes identified in the text mining phase. To do that, we downloaded the human sequences for the proteins known to contribute to either of these two pathways. We employed BLASTP to investigate the similarity between these human proteins and various species covering 1 billion years, starting *Trichoplax adhaerens*, common fruit fly (*Drosophila melanogaster*), roundworm (*Caenorhabditis elegans*), sea anemone (*Nematostella vectensis*), sea squirt (*Ciona intestinalis*), Lampreys (*Petromyzon marinus*), Zebrafish (*Danio rerio*), Elephant shark (*Callorhinchus milii*), red junglefowl (*Gallus gallus*), House mouse (*Mus musculus*) and chimpanzee (*Pan troglodytes*). In order to increase accuracy, we only selected the longest transcript for downstream analysis. We utilized a threshold of 1e-10 to accept identified proteins as putative candidates. Phylogenetic analysis was performed in three steps. First, we used Clustalw to align the investigated proteins in Seaview [24,25]. After that, we utilized ProtTest to determine the best amino acid replacement model as described previously [26,27]. Thirdly, we constructed the trees by using PHYML in Seaview with five random starts for each tree [28]. To measure functional specificity, we used the SDP server [29] and for functional conservation, we used ConSurf Server [30].

### 2.3. Ancestral Sequence Reconstruction (ASR)

We applied the maximum likelihood method to infer the ancestral sequence of each of the proteins investigated. For each protein, we used the ASR algorithm implemented in MEGA6 to build ancestral sequences [31]. This was followed by BlastP against the nearest earlier diverging organism. BlastP outcome was only accepted if the *E*-value threshold was less than e^−10^.

### 2.4. HHsearch

HHsearch method was used to examine the evolutionary history of PECAM1. Only proteins that have already diverged before PECAM1 were considered as candidate parents [27].

## 3. Results

**Figure 1:**
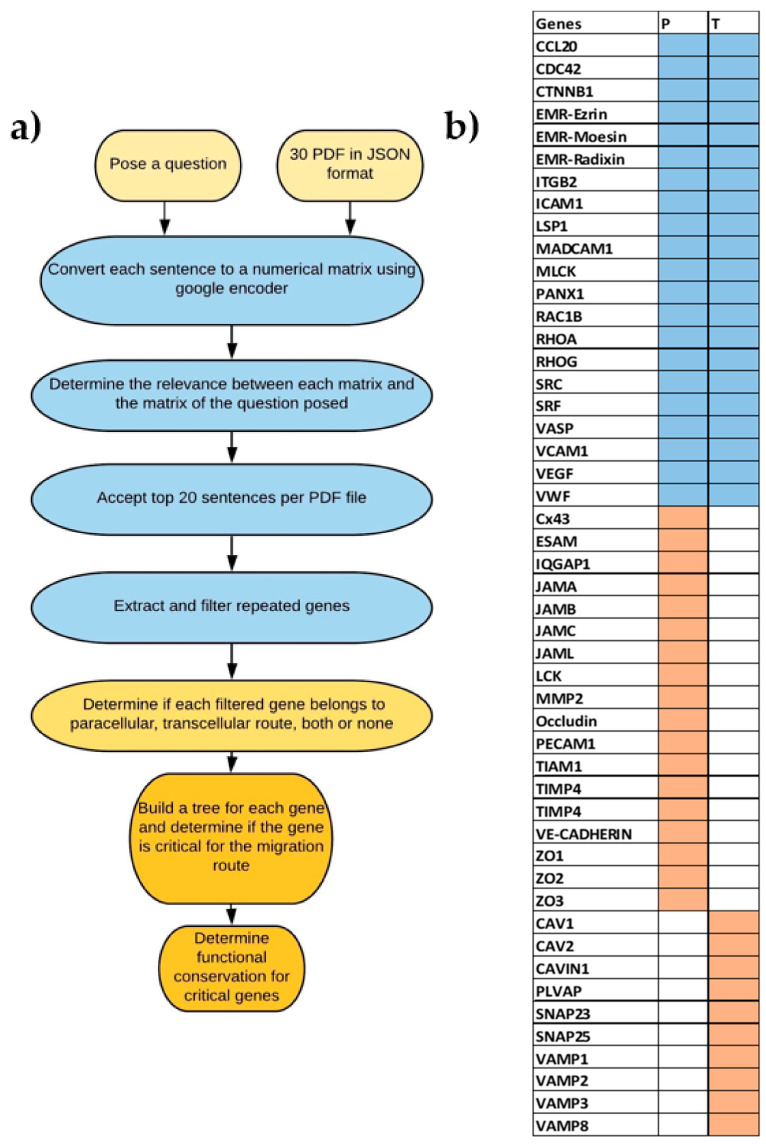
Workflow used in this report. (**a**) AI text mining procedure was done in various phases. First, answers for the imposed question were collected. Secondly, answers were filtered and gene names were extracted. Thirdly, the resulting genes were clustered according to their reported function into a paracellular pathway component, transcellular component, or both. Fourthly, phylogenetic analysis was done and lastly, functional conservation for critical genes was performed. (**b**) The genes resulting from the AI step were categorized based on literature review into one of three groups; shared genes (in navy blue) and paracellular (P) and transcellular (T) (in light red).

Our results indicate that paracellular and transcellular migration routes have different but interconnected evolution history. We applied an AI in-house system tool (AIST) to extract genes that are expressed by endothelial cells and can be affecting the diapedesis process migration. We identified genes that are common between the two systems as well as genes that are exclusively active in one system but not the other and are critical for the diapedesis process such as PECAM1 in paracellular transmigration and CAV1 in transcellular transmigration. From that, we built phylogenetic trees to examine the history of divergence of these genes. Our results indicate that both systems have components that are ancient being expressed in lower invertebrates. However, critical components responsible for the transmigration route (e.g., CAV1) have diverged earlier than their counterparts of the paracellular route (PECAM1). As earlier invertebrates do not have immune cells, CAV1 might have evolved to digest food. Whereas, HH search and ancestral sequence reconstruction show that PECAM1 evolved from FCRL5 which is known to act as a mechanical sensor. Both systems coexist in vertebrates. The lymphocytes’ choice of route of migration could be related to the interaction of the mechanical forces between these cells and the surface of the endothelial cells. Taken together our results highlight the significant differences in the evolutionary history between the two diapedesis processes and shed more light on an answer to a long-disputed question. We used an in-house AI text mining approach to locate the genes associated with either paracellular or trans-cellular migration (Figure 1a). We employed automatic literature review results using our in house AIST system. Our system was able to locate more than 99 genes. We validated the accuracy of our model by performing manual search for the identified genes (Supplementary File S1). 50 genes were manually filtered as they were associated with the lymphocytes infiltrating the brain or with cellular process to directly linked to paracellular or trans cellular migration routes We manually categorized the remaining genes (49 genes, Supplementary File S2) based in their reported abilities into one of the two groups (Figure 1b). The number of paracellular migration-associated genes was 39, while the number of transcellular migration-associated genes was 31. The number of genes associated with both migration schemes was 21.

The genes that were identified to play an active role within the paracellular migration route were Cx43, CLDN5, Esam, Iqgap1, JamA, JamB, JamC, Jaml, Lck, Mmp2, Occludin, Pecam1, Tiam1, Timp1, Timp4, VE-Cadherin, Zo1, Zo2, and Zo3 (Figure 1b). These genes could be grouped into three main groups (i) genes that are expressed in the space between cells. This group’s main function is to protect the integrity of the blood-brain barrier, and it includes Zo1, Zo2, Zo3 known as Zonula occludens. Moreover, JAMs, including JAMA, JAMB, JAMC, and JAML (junctional adhesion molecule), belong to this group. Additionally, Cx43, Claudin, Occludin, Esam, and VE-CADHERIN are localized in the same area [32,33]. (ii) The second group is important in regulating the movement of the cells through the space between the endothelial cells. This group includes PCAM1and MMP2. For example, MMP2 degrades collagen and gelatin of the extra-cellular matrix known to act as anchors of the endothelial cells. (iii) The third group includes genes that regulate the genes of the previous two groups. For example, Iqgap1 regulates the function of claudin (e.g., CLDN5), while LCK regulates the function of PECAM1 [34,35]. Timp 1 and Timp4 regulate Mmp2 [36,37].

Results of the text mining process highlighted various genes that are known to be specifically involved in the transcellular migration, including; CAV1, CAV2, CAVIN1, PLVAP, SNAP23, SNAP25, VAMP1, VAMP2, VAMP3, and VAMP8 (Figure 1b). Cav1, Cav2, and CAVIN1 are responsible for the construction of the caveolae, which is a hallmark of transcellular lymphocyte migration [38,39]. Plvap is also a structural component of caveolae and is involved in transcellular transport [40]. The next group of genes’ main function is to promote plasma membrane fusion among different cells. This group includes; Vamp2, Vamp3, Vamp8, Snap23, and Snap25. Vamp2, Vamp3, Vamp8 belong to the VAMPs family (Vesicle-associated Membrane Protein) fusogenic proteins, while Snap23 and Snap25 belong to the SNAP25 family [41,42,43].

**Figure 2:**
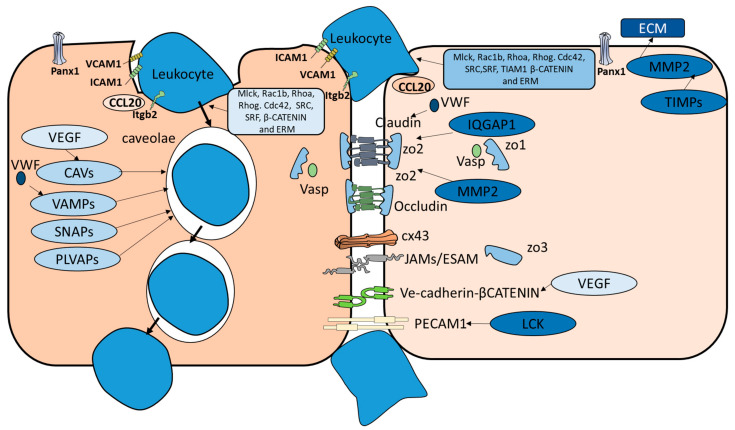
Paracellular versus transcellular migration pathways. Several genes are used exclusively by paracellular diapedesis route such as CX43, Claudin, Occludin, JAMs and VE-cadherin. Similarly, CAVs and VAMPs seem to be activated only in transcellular pathway. Genes that are activated in both pathways include those who function in transmembrane movements’ such as RhoA and β-catenin pathway.

Our AITS system detected various genes that were reported to be active in both transcellular and paracellular diapedesis routes (Figure 2). These genes included; (i) genes that are responsible for the recruitment of lymphocytes such as CCL20 [2]. (ii) Genes that are critical for lymphocytes adhesion to endothelial cells such as ICAM1, VCAM1, and Madcam1 [44,45]. (iii) Genes that promote cytoskeleton movement and constitute an important element of cross-talk between the two migration routes. These genes include; Mlck, Rac1b, Rhoa, Rhog. Cdc42. ITGB2, SRF and ERM family (ezrin/radixin/moesin) [46,47,48,49] (iv) Genes that enhance immune cells infiltration of endothelial cells in both paracellular and transcellular routes such as Panx1 [50,51]. (v) Genes that play different roles in paracellular and transcellular routes and these genes include LSP1 which is an endothelial F-actin binding protein, and it moves from the nucleus to the cytosol and is associated with the cytoskeleton on stimulation and the formation of the dome structures [52]. VEGF interacts with VE-cadherin in the case of paracellular migration and upregulates CAV1 in the case of transcellular migration [53,54]. VWF is a glycoprotein that is only synthesized by endothelial cells and megakaryocytes. VWF plays an inhibitory role in Claudin-5 expression in paracellular transmigration and VAMP3 in the case of transcellular migration [42,55] (Figure 1b) [56,57].

### 3.1. Phylogenetic Analysis of Components of the Diapedesis Mechanism

**Figure 3:**
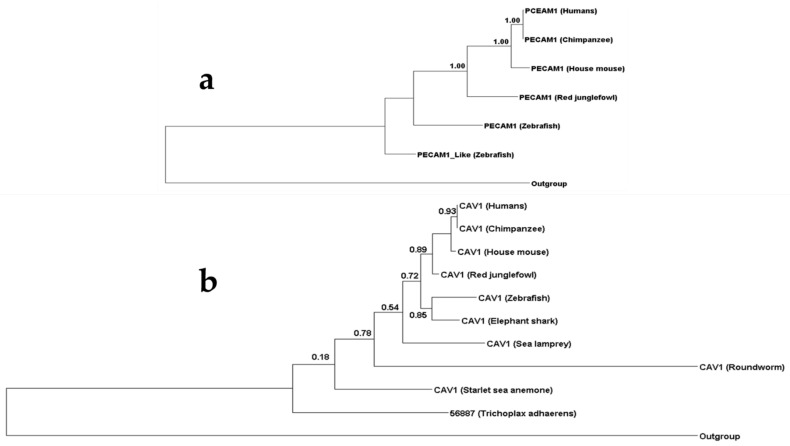
Comparison between the phylogenetic history of the active components of paracellular and transcellular migration pathways. We investigated the phylogenetic of (**a**) PECAM1 the main active component of paracellular migration and (**b**) CAV1 the main active component of the transcellular migration pathway. PECAM1 diverged during bony fish emergence where CAV1 diverged during placozoa first appearance.

Our results revealed that the evolutionary history of paracellular and transcellular migration routes is different but with various connecting points. Several genes contributing to both migration routes appear to be ancient. RAC1B, RHOA, RHOG, SRC, SRF VASP, CDC42, CTNNB1, ERMs, ITGB2 and VWF appear in lower invertebrates, such as *Trichoplax adhaerens* as well as *Nematostella vectensis*. However, both systems also utilize more recent diverging genes such as ICAM1, VCAM1, LSP1, MADCAM1, and CCL20. Interestingly, most genes that are only known to contribute to the paracellular migration route, are relatively recent, such as PECAM1 (Figure 3a) and ESAM that diverged during *Danio rerio* emergence. JAMs diverged during elephant shark emergence, while Occludin first appeared in lampreys. Although several genes that are specific for paracellular route seem to be ancient such as Zonula occludens. Zonula occludens have at least one ortholog in *Nematostella vectensis* and *Trichoplax adhaerens* but do not appear in other investigated invertebrates such as roundworm and fruit fly. Intriguingly, most of the genes that are specific for the transcellular route seem ancient, such as CAV1 (Figure 3b), CAV2, SNAP23, SNAP25, VAMP1, VAMP2, VAMP3, and VAMP8 and have at least one ortholog in both *Nematostella vectensis* and *Trichoplax adhaerens* with the exception of CAVIN1 and PLVAP.

**Figure 4:**
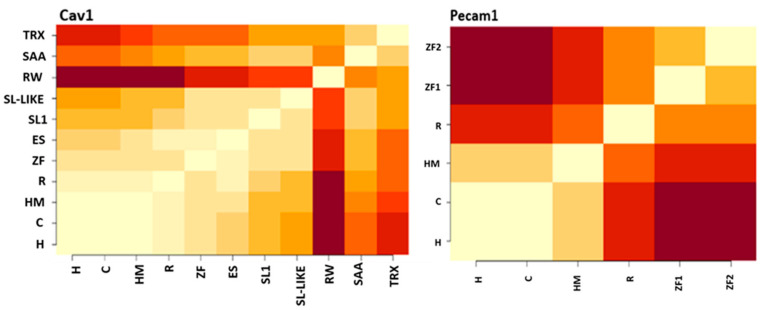
Comparative functional conservation between PECAM1 and CAV1. Pecam1 and CAV1, have low similarity. Symbols used are; Humans (H), Chimp (C), HM (house mouse), R (Red Jungle fowl), ZF (Zebrafish), ES (Elephant shark), (RW) Roundworm, Starlet sea anemone (SAA), SL (Sea lampreys) and TRX(Trichoplax).

Interestingly, we found that neither PECAM1 or CAV1 are highly conserved. This was evident by identity comparison, where the lowest value between PECAM1 species was 24% for CAV1, it was 29% (Figure 4). This is further conformed by the percentage of conserved functional sites, where in case of PECAM1 (16%), and in case of CAV1 it is 17% (Figure 4). This could be expected in case of CAV1 was expressed among various organism from *Trichoplax* to humans. Notably the low conservation of PECAM1 gene is further confirmed with high number of functional specificity residue of PECAM1 between the terrestrial species and the marine species (Supplementary File S3).

This is also mirrored in functional conservation of residue in both PECM1 and CAV1, where highly conserved ratios of residues are 16.5% and 18.4% respectively.

### 3.2. PECAM1 Evolved to Function as a Mechanical Sensor

**Figure 5:**
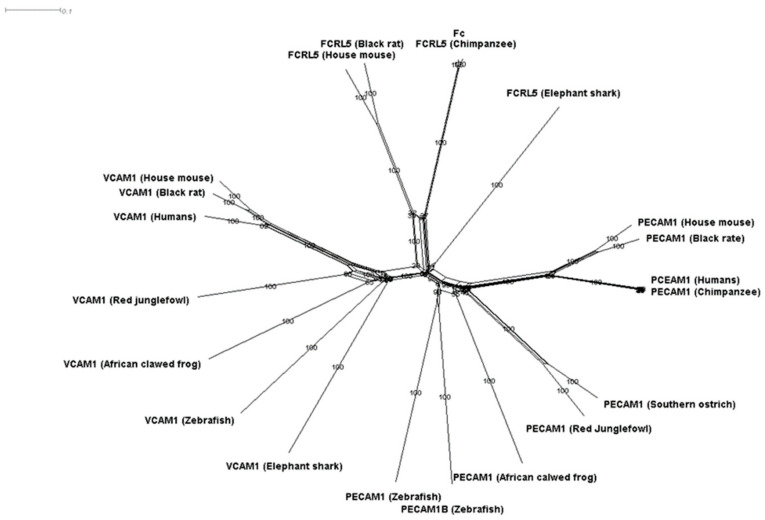
Evolutionary network of PECAM1. We used HHserach, Blastp, and Splittrees to build an evolutionary network for PECAM1. We detected two putative ancestors for PECAM1 (VCAM1 and FCRL5). However, FCRL5 has the shortest evolutionary pathway to PECAM1.

Our results indicated that PECAM1 evolved to be a mechanical sensor. We constructed the ancestral sequence of this gene using Mega6 and used blast and HHsearch to locate its probable ancestral orthologs. Our results identified FCRL5 and VCAM1 to be putative ancestral candidates. After that, we used SplitTress to build the most probable network of evolution for these three genes (Figure 5). Interestingly, FCRL5 is known for its ability to mediate B cells to sense Ig quality and engage with them. B cells were shown to use mechanical energy to test antigen bonding strength [58]. These results highlight mechanical sensing as a putative function for PECAM1.

**Figure 6:**
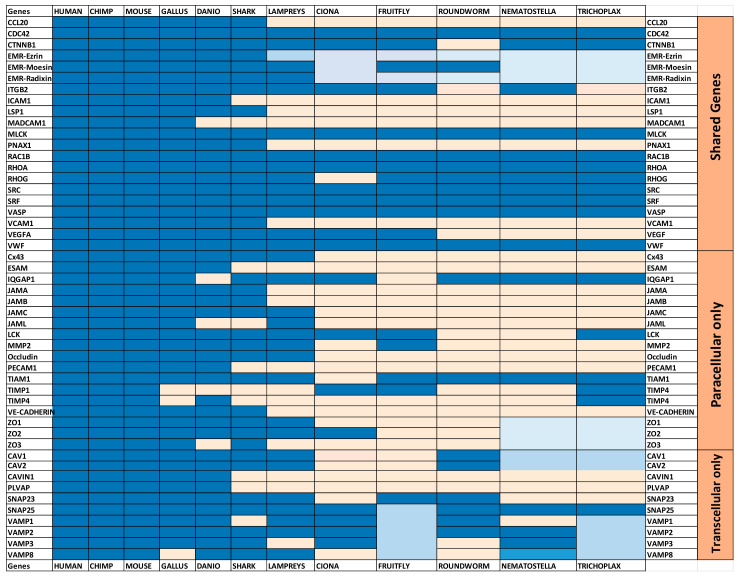
Phylogenetic history of the main components of paracellular and transcellular migration. Dark blue represents homologs found in respective genes. Light blue represents orthologs found for a gene family. Light red represents non existing orthologs or homologs. Almost all exclusive transcellular route genes identified are ancient except for CAVLIN1 and PLVAP, which both first appear in *Danio rerio*. Most genes that are exclusively active in the paracellular route have recently diverged such as CX43, JAMs, JAML, Occludin, PECAM1, VE-cadherin. Genes that are active in both routes are mostly ancient such as RhoA, RhoG, SRC, SRF VASP, CDC42, CTNNB1 and VWF.

Our results indicate that both paracellular and transcellular migration routes coevolved with immune cells. In the early metazoans, regulating transmembrane motion was one of the first mechanisms to evolve as evident by the existence of RhoA and Rac1b as well as Rhog in *Trichoplax adhaerens*, *Nematostella vectensis*, and *C. elegans* (Figure 6). CAV1 also appeared during the same period (Table 1 and Table 2). Probably CAV1-mediated mechanisms evolved to help digest food as there are no immune cells in *Trichoplax adhaerens* (Table 1). A proper blood-brain barrier starts to exist in *Drosophila melanogaster* and with it appeared genes that are responsible for maintaining BBB integrity as well as immune cells-like known as hemocytes (Table 1). Lampreys evolved with their specific phenotypes of immune cells such as VLAR, VLARB, and VLARC. The main protein controlling diapedesis in lampreys is not yet known. Starting from *Callorhinchus milii* (elephant shark), both systems of diapedesis started to formally exist in line with the emergence of lymphocytes.

## 4. Discussion

In this investigation, we show that the evolutionary history of transmigration’s main methods is different. Although the two methods share key mechanisms that help permit transmigration through the endothelial layer of the BBB, they have various components that have different functions and different evolutionary histories. These key components include CAV1 and PECAM. CAV1 which is a critical contributor in transcellular migration has diverged during *Trichoplax adhaerens* and continued to be expressed in major lines except for *Drosophila* and *Ciona*. PECAM1 on the other hand is relatively new as it diverged during the emergence of cartilaginous fish such as the elephant shark. Taken together this report indicates that contrary to the ongoing belief of the resemblance in nature and function of paracellular and transcellular migration, they constitute two divergent processes.

### 4.1. Origin of Transmigration Routes

Although paracellular and transcellular migration mechanisms contain various identical components, the two systems constitute two divergent evolutionary pathways. In vertebrates, both systems are dependent on ICAM1 and VCAM1 interaction with the invading leukocytes along with the binding of CCR7 expressed on lymphocytes and CCL21 produced by endothelial cells [59]. Our analysis shows that ICAM1 and VCAM1 and CCL20 diverged during the bony fish and cartilaginous fish respectively (Figure 6). Additionally, both systems use the RhoA pathway to initiate membrane movement. RhoA is an ancient gene that is likely to have diverged before the emergence of *Trichoplax* [60]. RhoA initiates the phosphorylation of VE-Cadherin in the paracellular extravasation process [61]. In transcellular migration, RhoA interacts with CAV1 to promote signal sensing. It is important to note that CAV1 and its main interactors (e.g., VAMP1) diverged during the emergence of Trichoplax (Figure 6). On the other hand, PECAM1 which has diverged during the *Danio rerio* emergence likely from FCLR5 (Figure 5). PECAM1 is capable to form homophilic bonds and act as a mechanical sensor to regulate the movement of the cell membrane [62]. Several studies support our findings that these two processes constitute two different processes. First, it was reported that lymphocytes infiltrating the endothelial cells treated with anti PECAM1 favor transcellular migration [9]. Secondly, it has been shown that CAV1^−/−^ mice almost exclusively used paracellular migration [63]. Thirdly, it was reported that PECAM1 and CAV1 do not colocalize together in one cellular compartment [64]. Furthermore, PECAM1 and VE-Cadherin form a mechano-sensing complex that is ligand-specific. This complex transfers mechanical force exerted by the incoming leukocytes on the endothelial cell surface [65]. This is further confirmed by the function of Tiam1 which is expressed on T cells as it plays an important role in controlling cell polarity. Interestingly, T cells that lack *Tiam1* largely follow *transcellular* migration [66]. In Parallel, CAV1 also acts, as a mechanical sensor that regulates proliferation through the endothelial walls. This evidence and findings suggest that PECAM1 led and CAV1 led migration pathways have evolved to accommodate two types of mechanical forces. This conclusion is further supported by the observation that CAV1 promotes CD4+ Th1 and Treg infiltration of the brain as opposed to CD4+ Th17. Interestingly, in case of PECAM1 deficient mice, Th17 cells follow a transcellular route [9]. Conversely, in case of CAV1 KO mice, Th1 follows a paracellular route. CD4+ T cells’ migration and movement are dependent on the cell’s desire to achieve the lowest strain energy state possible [67,68]. Achieving the lowest strain energy state is done by investigating the environment through its parts (filopodia and lamellipodia) to identify the lowest mechanical resistance to the cellular movement [69]. Due to the difference in nature of the cellular construction between paracellular and transcellular routes, the resistance forces exerted on migrating lymphocytes are different. Th1 cells have been shown to favor transcellular migration while Th17 cells prefer the paracellular route [9,22]. These observations indicate that in the case of Th1, the lowest mechanical resistance route is transcellular, conversely for Th17 cells, the lowest resistance route is paracellular. The reason behind these observations could lie in the shape and size of the two cells. It has been reported that Th17 size is 9.8 μm, while Th1 size is 10.4 μm. Another aspect that could be contributing to the difference in resistance routes is the protein-protein interactions experienced by each cell. PECAM1 controls the paracellular route through forming homodimers with PECAM1 expressed on T cells [9]. CAV1 forms caveolae allowing Th1 transcellular migration. However, other protein-protein interactions could also be contributing to forming these resistive forces. This hypothesis is further supported by the observation that knocking out the lymphocytes Llfa1 gene which is responsible for the interaction with the endothelial cells during the first phase of transmigration resulted in deformations and changing the biomechanical aspects of Th1, including effects on their filopodia and lamellipodia, but not Th17 [69]. We postulate that single cell RNA-seq analysis could shed more light on CD4+ T cells heterogeneous interactions with the endothelial cells of the BBB [70]. To summarize, PECAM1 and CAV1 which are responsible for regulating the formation of the space for migration in transcellular and paracellular migration receptively are mutually exclusive mechanosensing and have different phylogenetic origin and history.

### 4.2. Transmigration in Lower Invertebrates

Lower invertebrates (e.g., *Trichoplax adhereans* and Nematostella vectensis, in addition to Caenorhabditis elegans) constitute a phylogenetic paradox. No immune cells have been reported in them (Table 1) [71,72,73]. Trichoplax does not show a rudimentary brain and hence no blood-brain barrier. *Nematostella* vectensis shows only two centralized neural pathways (WNT and BMP), but lack brain-like structures [74,75]. They also express several genes related to astrocytes that play an important role in the BBB, such as Rap1b, RhoA, Vamp1, and Zo1(Figure 6). *C. elegans* have central neurons in a brain-like structure and a BBB like structure that expresses claudin (Figure 6) [74]. Interestingly, all three species express CAV1 that is responsible for forming caveolae (Figure 6). Caveolae are not expressed in fungi and plants thus it seems to be a metazoan invention [76]. In an interesting study, CAV1 expressed in bacteria initiated the formation of caveolae [77]. However, these reports have not been yet repeated in any of the lower invertebrates. The question arises about the need for the expression of CAV1 in these species. One hypothesis could be that caveolae were initially used to transfer macromolecules as well as digestion. This argument is further supported by other reports that showed that Trichoplax expresses CAV molecules to digest algae [78]. In *C. elegans*, caveolae have yet been detected [79]. It was suggested that a passive diffusion trans-endothelial of macromolecules is used [80]. CAV1 plays various roles in *C. elegans* including neurotransmission [81]. However, if it contributes toward the regulation of passive diffusion of molecules into the brain is not yet known.

### 4.3. Transmigration in Insects

Our results indicate that transmigration is likely to follow a paracellular route in insects. This hypothesis is supported by multiple pieces of evidence. First Drosophila does not express CAV1 (Figure 6) making it impossible for *Drosophila melanogaster* to form caveolae [79]. Interestingly, Drosophila exhibits a distinct complex structure that resembles the BBB, with an important role for claudin and septate junctions, albeit the drosophila BBB main building blocks are formed of glia such as subperineurial glia (SPG), perineurial glia, and neural lamella [82]. Furthermore, immune cells extravasate from vessels to wounds [83]. Taken together, our reports suggest that paracellular transmigration of immune cells from drosophila hemolymph to its CNS through the adherens junctions and the septate junctions of the BBB, albeit the key mechano-sensors elements, are yet to be found.

### 4.4. Transmigration in Lampreys

How diapedesis takes place in lampreys is still an open question. The lampreys have a developed brain [84]. Lampreys brain contains areas necessary for controlling advanced emotions such as the hippocampus and hypothalamus [84]. They are also capable of showing advanced cognitive abilities [85]. Three adaptive immune cells lineages have been localized in lamprey’s genome, (e.g., VLRA, VLRB, and VLRC), which are analogous to T cells and B cells in vertebrates. Lampreys exhibit an endothelial BBB [86]. The mechanism of extravasation of immune cells in lampreys is yet to be found. Occludin, as well as ZO1 and ZO2, and JAMC, are expressed in lamprey, which indicates a degree of BBB integrity. Interestingly, we could not locate PECAM1 lamprey’s genome neither did we locate VE-cadherin or I-CAM1 (Figure 6), however, CAV1 is highly conserved in lampreys (Figure 6). Interestingly VAMP1, VAMP2 which are critical for CAV1 function are also conserved. These findings can support the hypothesis the lampreys use predominantly, transcellular routes.

### 4.5. Functional Aspects of the Findings

Regulation of the BBB Infiltration by immune cells constitutes a novel therapy in several neuropathological diseases such as multiple sclerosis. Pathogenic immune cells infiltration of the brain plays a critical role in the genesis and prognosis of various neuropathological conditions. Under normal conditions the BBB is highly integral and thus prevents immune cells from migrating to the brain. However, under neuropathological conditions, the BBB loses its integrity and immune cells exploit this property and increase their infiltration to the brain, thus exacerbating pathological conditions [87]. Th17 cells are known to contribute to various neuropathological symptoms such as neural damage, demyelination, and lesion formation [88]. Current therapeutic strategies targeting multiple sclerosis aim at preventing Th17 from infiltrating the brain. These strategies are implemented by targeting the integrin α4β1 expressed on T cells and connected to VCAM1 expressed on the endothelial cells [2,3]. However, using drug therapies based on these strategies increases the risk of other diseases. For example, Natalizumab augments the possibility of causing fatal progressive multifocal leukoencephalopathy (PML) as well as other viral diseases [89]. Thus, novel alternative strategies are critically needed. Th17 cells were shown to specifically favor paracellular transmigration [9]. We postulate in this report that, paracellular transmigration is mainly controlled by PECAM1, while CAV1 is fundamental for transcellular migration. Interestingly, in PECAM1 KO mice, Th17 cells switch to a transcellular migration route [9]. Thus, to prevent Th17 cells from infiltrating the brain, a strategy based on targeting PECAM1 and CAV1 could constitute a viable approach. Furthermore, Tregs, are known to regulate Th17 function. However, they have a low ability to infiltrate the brain. Studying Tregs migration route would shed more light on possible drug targets that could increase natural regulation of pathogenic cells such as Th17.

## 5. Conclusions

Paracellular and transcellular migration mechanisms have different origins. Transcellular migration based on caveolae existence seems to have started during *Trichoplax* emergence. Paracellular migration appeared during *Drosophila melanogaster* divergence. Both mechanisms evolved using different components such as VAMP1 in the case of Transcellular and VE-cadherin in the case of paracellular diapedesis route. Our report indicates that in lampreys the dominant mode of transmigration could be transcellular, while we predict that the dominant mode of transmigration in Drosophila melanogaster is paracellular, based on the lack of CAV1 expression. During the emergence of cartilaginous fish, both systems started to appear side by side.

## Figures and Tables

**Table 1:** Comparison of the evolution history of diapedesis mechanisms by interacting components.

Species	Brain Structure	Immune Cells	BBB	Critical Component		Known Diapedesis Mechanism
				Cav1	Pecam1	
*T. adhaerens*	No brain detected	No known immune cells	No	Yes	no	NA
*N. vectensis*	Two brain pathways observed	Ameboyctes	unknown	Yes	no	NA
*C. elegans*	Brain-like structure	No adaptive immune system or mobile immune cells identified	glia	Yes	No	NA
*D. melanogaster*	Brain-like structure	Hemocytes	septal	no	no	Likely Paracellular
*C. intestinalis*	177 neurons identified	Hemocytes	unknown	Yes	No	Likely transcellular
*P. marinus*	Brian structure exits	VLAR, VLARB and VLARC cells	Yes	Yes	No	Likely transcellular
Marine vertebrates	Brian structure exists	Adaptive and innate system developed	yes	Yes	Yes	Transcellular and paracellular
Terrestrial vertebrates	Brian structure exists	Adaptive and innate system developed	yes	Yes	Yes	Transcellular and paracellular

**Table 2:** Comparison of the evolution history of diapedesis mechanisms by known important genes.

Species	In Both Migration Routes	Paracellular Only	Transcellular Only
*T. adhaerens*	CDC42, CTNNB1, MLCK, RAC1B, RHOA, RHOG, SRC, SRF, VASP and VWF	IQGAP1, LCK, TIAM1, TIMP1 and TIMP4, ZO orthologs	CAV orthologs, VAMP orthologs, and SNAP orthologs
*N. vectensis*	CDC42, CTNNB1, MLCK, RAC1B, RHOA, RHOG, SRC, SRF, VASP and VWF	IQGAP1, TIAM1, ZO orthologs	CAV orthologs, VAMP orthologs, and SNAP orthologs
*C. elegans*	CDC42, RAC1B, RHOA RHOG, SRC, SRF, VASP, MLCK and VWF	IQGAP1, TIAM1	CAV orthologs, VAMP orthologs, and SNAP orthologs
*D. melanogaster*	CDC42, CTNNB1, MLCK, RAC1B, RHOA, RHOG, SRC, SRF, VASP, VEGFA, and VWF	LCK, MMP2, TIAM1 and TIMP1	VAMP orthologs and SNAP orthologs
*C. intestinalis*	CDC42, CTNNB1, MLCK RAC1B, RHOA, SRC, SRF, VASP, VEGFA, VWF and CLDN5	IQGAP1, LCK, TIMP1 and ZO2	CAV orthologs, VAMP orthologs, and SNAP orthologs
*P. marinus*	CDC42, CTNNB1, MLCK, RAC1B, RHOA, RHOG, SRC, SRF, VASP, VEGFA and VWF	CLDN5, Cx43, IQGAP1, JAMC, JAML, LCK, MMP2, Occludin, TIAM1, ZO1, ZO2	CAV orthologs, VAMP orthologs, and SNAP orthologs
Marine vertebrates	CCL20, CDC42, CTNNB1, ICAM1, LSP1, MLCK, PANX1, RAC1B, RHOA, RHOG, SRC, SRF, VASP, VCAM1, VEGFA, and VWF	CLDN5, Cx43, ESAM, JAMA, JAMB, JAMC, LCK, MMP2, Occludin, TIAM1, VE-CADHERIN, ZO1, ZO2	CAV1, CAV2, SNAP23, SNAP25, VAMP2, VAMP3, VAMP8
Terrestrial vertebrates	CCL20, CDC42, CTNNB1, ICAM1, LSP1, MADCAM1, MLCK, PANX1, RAC1B, RHOA, RHOG, SRC, SRF, VASP, VCAM1, VEGFA and VWF	CLDN5, Cx43, ESAM, IQGAP1, JAMA, JAMB, JAMC, JAML, LCK, MMP2, Occludin, PECAM1, TIAM1, TIMP1, TIMP4, VE-CADHERIN, ZO1, ZO2, ZO3	CAV1, CAV2, CAVIN1, PLVAP, SNAP23, SNAP25, VAMP1, VAMP2, VAMP3, VAMP8

## Supplementary Materials

The following Supplementary Materials are available online at https://www.mdpi.com/2073-4425/12/2/254/s1, Supplementary File S1: Validation of the results of the AI system, Supplementary File S2: Human fasta files, Supplementary File S3: Results of the functional specificity analysis.
