# Supplementary material for: Paracellular and Transcellular Leukocytes Diapedesis Are Divergent but Interconnected Evolutionary Events
Source: Genes (Basel). 2021 Feb 10;12(2):254. doi: 10.3390/genes12020254 (PMC7916592; doi:10.3390/genes12020254)
Supplement: Supplementary file 1 [file genes-12-00254-s001.zip › suplementary_3adera/supplemantary_file1_model_validation_3adera.docx]

| **Supplementary file (3): Validation of the function of the genes identified by AIS tool** | | | | | | |
| --- | --- | --- | --- | --- | --- | --- |
| **Genes** | **P** | **T** | **Evidence** | **Disease** | **Reference** | **tissue** |
| **CCL20** |  |  | Upregulated | Multiple sclerosis | [1][2] | Endothelial cells |
| **CDC42** |  |  | Upregulated | Cerebral Vascular Malformations | [3] | Endothelial cells |
| **CLDN1** |  |  | upregulated | Stroke, Multiple sclerosis | [4] | Endothelial cells |
| **CTNNB1** |  |  | upregulated | Multiple sclerosis | [5] | Endothelial cells |
| **ERM** |  |  | upregulated | streptococcal Fic | [6][7][8] | Endothelial cells |
| **ITB2** |  |  | Upregulated | ischemia | [9] | Endothelial cells |
| **ICAM1** |  |  | Upregulated | Multiple sclerosis | [10] | CSF |
| **LSP1** |  |  | Upregulated | (CCMs) Cerebral Cavernous Malformations | [11] | Endothelial cells |
| **MADCAM1** |  |  | Upregulated | IFNγ treated | [12] | Endothelial cells |
| **MLCK** |  |  | Upregulated | In vitro model | [13] | Endothelial cells |
| **PNAX1** |  |  | Upregulated | Stroke | [14] | Endothelial cells |
| **RAC1B** |  |  | Upregulated | Hyperglycemia | [15] | Endothelial cells |
| **RHOG** |  |  | Upregulated | In vitro | [16] | Endothelial cells |
| **RHOA** |  |  | Upregulated | Hyperglycemia | [15] | Endothelial cells |
| **SRC** |  |  | Downregulated | In vitro BBB model of MafA | [17] | Endothelial cells |
| **SRF** |  |  | Downregulated | stroke | [18] | Endothelial cells |
| **VASP** |  |  | upregulated | In vitro BBB model of hypoxia | [19] | Endothelial cells |
| **VCAM1** |  |  | Upregulated | Multiple sclerosis | [20] | Endothelial cells |
| **VEGF** |  |  | Upregulated | Brain injury | [21] | Endothelial cells |
| **VWF** |  |  | Upregulated | Single cell mouse brain (homeostasis) | [22] | Endothelial cells |

| **Cx43** |  |  | downregulated | Familial cerebral cavernous malformations type I | [23] | Endothelial cells |
| --- | --- | --- | --- | --- | --- | --- |
| **ESAM** |  |  | Association | schizophrenia | [24] | Endothelial cells |
| **IQGAP1** |  |  | Upregulated | In vitro brain injury model | [25] | Endothelial cells |
| **JAMA** |  |  | Downregulated | vivo model of BBB breakdown | [26] | Endothelial cells |
| **JAMB** |  |  | Downregulated |  |  | Endothelial cells |
| **JAMC** |  |  | Downregulated |  |  | Endothelial cells |
| **JAML** |  |  | upregulated | multiple sclerosis | [27] | Endothelial cells |
| **LCK** |  |  | Unknown | Shown to control PECAM1 function | [28] | Endothelial cells |
| **MMP2** |  |  | Upregulated | Hypertension/stroke/Alzheimer | [29] | Endothelial cells |
| **MMP9** |  |  | Upregulated | Hypertension/stroke/Alzheimer | [29] | Endothelial cells |
| **Occludin** |  |  | Downregulated | multiple sclerosis | [30] | Endothelial cells |
| **PANX1** |  |  | upregulated | ischemic stroke | [14] | Endothelial cells |
| **PECAM1** |  |  | Upregulated | multiple sclerosis | [31] | Endothelial cells |
| **TIAM1** |  |  | Unknown | Shown to be downstream target of VE-CADHERIN | [32] | Endothelial cells |
| **TIMP4** |  |  | Downregulated | In vitro response to Neisseria meningitidis | [17] | Endothelial cells |
| **TIMP1** |  |  | No change in qPCR, but shown to inhibit MMP2 | Focal Cerebral Ischemia | [33] | Endothelial cells |
| **VE-CADHERIN** |  |  | Downregulated | multiple sclerosis | [34] | Endothelial cells |
| **ZO1** |  |  | Downregulated | multiple sclerosis | [30] | Endothelial cells |
| **ZO2** |  |  | Downregulated | CCL2 treatment | [35] | Endothelial cells |
| **ZO3** |  |  | Unknown | Detectd in combination of ZO-1 and ZO-2 | [36] | Endothelial cells |

| **CAV1** |  |  | Downregulated | chronic traumatic encephalopathy | [37] | Endothelial cells |
| --- | --- | --- | --- | --- | --- | --- |
| **CAV2** |  |  | Upregulated | Traumatic brain injury | [38][39] | Endothelial cells |
| **CAVIN1** |  |  | Unknown | Shown to be needed for caveolae formation | [40] | Endothelial cells |
| **PLVAP** |  |  | Downregulated | cerebral edema | [41] | Endothelial cells |
| **SNAP23** |  |  | Unknown | Shown to primarily interact with VAMPs | [42] | Endothelial cells |
| **SNAP25** |  |  |  |  |  | Endothelial cells |
| **VAMP1** |  |  | Upregulated in adults | Time point embryo versus adult | [43] | Both left and right lateral ventricular choroid plexuses |
| **VAMP2** |  |  | Unknown | Shown to interact with CAV1 for caveolae formation | [44] | Endothelial cells |
| **VAMP3** |  |  |  |  |  |  |
| **VAMP8** |  |  | upregulated | Time point; embryo versus adult | [43] | Both left and right lateral ventricular choroid plexuses |

**References**

[1] L.-Y. Cui, S.-F. Chu, and N.-H. Chen, “The role of chemokines and chemokine receptors in multiple sclerosis,” *Int. Immunopharmacol.*, vol. 83, p. 106314, Jun. 2020, doi: 10.1016/j.intimp.2020.106314.

[2] Y. Arima *et al.*, “Regional Neural Activation Defines a Gateway for Autoreactive T Cells to Cross the Blood-Brain Barrier,” *Cell*, vol. 148, no. 3, pp. 447–457, Feb. 2012, doi: 10.1016/j.cell.2012.01.022.

[3] M. Castro *et al.*, “CDC42 Deletion Elicits Cerebral Vascular Malformations via Increased MEKK3-Dependent KLF4 Expression,” *Circ. Res.*, vol. 124, no. 8, pp. 1240–1252, Apr. 2019, doi: 10.1161/CIRCRESAHA.118.314300.

[4] N. Sladojevic *et al.*, “Claudin-1-Dependent Destabilization of the Blood–Brain Barrier in Chronic Stroke,” *J. Neurosci.*, vol. 39, no. 4, pp. 743–757, Jan. 2019, doi: 10.1523/JNEUROSCI.1432-18.2018.

[5] J. E. Lengfeld *et al.*, “Endothelial Wnt/β-catenin signaling reduces immune cell infiltration in multiple sclerosis,” *Proc. Natl. Acad. Sci. U. S. A.*, 2017, doi: 10.1073/pnas.1609905114.

[6] I. A. Romero, C. L. Amos, J. Greenwood, and P. Adamson, “Ezrin and moesin co-localise with ICAM-1 in brain endothelial cells but are not directly associated,” *Mol. Brain Res.*, vol. 105, no. 1–2, pp. 47–59, Sep. 2002, doi: 10.1016/S0169-328X(02)00392-3.

[7] Y. Hoshi *et al.*, “Distinct roles of ezrin, radixin and moesin in maintaining the plasma membrane localizations and functions of human blood–brain barrier transporters,” *J. Cereb. Blood Flow Metab.*, 2020, doi: 10.1177/0271678X19868880.

[8] Z. Ma *et al.*, “A streptococcal Fic domain-containing protein disrupts blood-brain barrier integrity by activating moesin in endothelial cells,” *PLOS Pathog.*, vol. 15, no. 5, p. e1007737, May 2019, doi: 10.1371/journal.ppat.1007737.

[9] E. Chavakis *et al.*, “Role of β2-integrins for homing and neovascularization capacity of endothelial progenitor cells,” *J. Exp. Med.*, 2005, doi: 10.1084/jem.20041402.

[10] M. K. Sharief, M. A. Noori, M. Ciardi, A. Cirelli, and E. J. Thompson, “Increased levels of circulating ICAM-1 in serum and cerebrospinal fluid of patients with active multiple sclerosis. Correlation with TNF-α and blood-brain barrier damage,” *J. Neuroimmunol.*, vol. 43, no. 1–2, pp. 15–21, Mar. 1993, doi: 10.1016/0165-5728(93)90070-F.

[11] S. Subhash *et al.*, “Transcriptome-wide Profiling of Cerebral Cavernous Malformations Patients Reveal Important Long noncoding RNA molecular signatures,” *Sci. Rep.*, vol. 9, no. 1, p. 18203, Dec. 2019, doi: 10.1038/s41598-019-54845-0.

[12] S. A. Sonar, S. Shaikh, N. Joshi, A. N. Atre, and G. Lal, “IFN‐γ promotes transendothelial migration of CD4 ^+^ T cells across the blood–brain barrier,” *Immunol. Cell Biol.*, vol. 95, no. 9, pp. 843–853, Oct. 2017, doi: 10.1038/icb.2017.56.

[13] P. V. Afonso *et al.*, “Human Blood-Brain Barrier Disruption by Retroviral-Infected Lymphocytes: Role of Myosin Light Chain Kinase in Endothelial Tight-Junction Disorganization,” *J. Immunol.*, vol. 179, no. 4, pp. 2576–2583, Aug. 2007, doi: 10.4049/jimmunol.179.4.2576.

[14] M. E. Good *et al.*, “Endothelial cell Pannexin1 modulates severity of ischemic stroke by regulating cerebral inflammation and myogenic tone,” *JCI Insight*, vol. 3, no. 6, Mar. 2018, doi: 10.1172/jci.insight.96272.

[15] S. Rom, N. A. Heldt, S. Gajghate, A. Seliga, N. L. Reichenbach, and Y. Persidsky, “Hyperglycemia and advanced glycation end products disrupt BBB and promote occludin and claudin-5 protein secretion on extracellular microvesicles,” *Sci. Rep.*, vol. 10, no. 1, p. 7274, Dec. 2020, doi: 10.1038/s41598-020-64349-x.

[16] J. D. Van Buul *et al.*, “RhoG regulates endothelial apical cup assembly downstream from ICAM1 engagement and is involved in leukocyte trans-endothelial migration,” *J. Cell Biol.*, 2007, doi: 10.1083/jcb.200612053.

[17] E. Káňová *et al.*, “Transcriptome analysis of human brain microvascular endothelial cells response to Neisseria meningitidis and its antigen MafA using RNA-seq,” *Sci. Rep.*, 2019, doi: 10.1038/s41598-019-55409-y.

[18] C. Weinl *et al.*, “Endothelial depletion of murine SRF/MRTF provokes intracerebral hemorrhagic stroke,” *Proc. Natl. Acad. Sci.*, vol. 112, no. 32, pp. 9914–9919, Aug. 2015, doi: 10.1073/pnas.1509047112.

[19] B. Davis *et al.*, “Role of vasodilator stimulated phosphoprotein in VEGF induced blood–brain barrier permeability in endothelial cell monolayers,” *Int. J. Dev. Neurosci.*, vol. 28, no. 6, pp. 423–428, Oct. 2010, doi: 10.1016/j.ijdevneu.2010.06.010.

[20] R. Allavena, S. Noy, M. Andrews, and N. Pullen, “CNS Elevation of Vascular and Not Mucosal Addressin Cell Adhesion Molecules in Patients with Multiple Sclerosis,” *Am. J. Pathol.*, vol. 176, no. 2, pp. 556–562, Feb. 2010, doi: 10.2353/ajpath.2010.090437.

[21] E. Papavassiliou *et al.*, “Vascular endothelial growth factor (vascular permeability factor) expression in injured rat brain,” *J. Neurosci. Res.*, vol. 49, no. 4, pp. 451–460, Aug. 1997, doi: 10.1002/(SICI)1097-4547(19970815)49:4<451::AID-JNR6>3.0.CO;2-7.

[22] M. B. Chen *et al.*, “Brain Endothelial Cells Are Exquisite Sensors of Age-Related Circulatory Cues,” *Cell Rep.*, vol. 30, no. 13, pp. 4418-4432.e4, Mar. 2020, doi: 10.1016/j.celrep.2020.03.012.

[23] A. M. Johnson *et al.*, “Connexin 43 gap junctions contribute to brain endothelial barrier hyperpermeability in familial cerebral cavernous malformations type III by modulating tight junction structure,” *FASEB J.*, vol. 32, no. 5, pp. 2615–2629, May 2018, doi: 10.1096/fj.201700699R.

[24] J. G. Pouget *et al.*, “Genome-wide association studies suggest limited immune gene enrichment in schizophrenia compared to 5 autoimmune diseases,” *Schizophr. Bull.*, 2016, doi: 10.1093/schbul/sbw059.

[25] S. Ikeda *et al.*, “IQGAP1 regulates reactive oxygen species-dependent endothelial cell migration through interacting with Nox2,” *Arterioscler. Thromb. Vasc. Biol.*, 2005, doi: 10.1161/01.ATV.0000187472.55437.af.

[26] D. Yeung, J. L. Manias, D. J. Stewart, and S. Nag, “Decreased junctional adhesion molecule-A expression during blood–brain barrier breakdown,” *Acta Neuropathol.*, vol. 115, no. 6, pp. 635–642, Jun. 2008, doi: 10.1007/s00401-008-0364-4.

[27] J. I. Alvarez, H. Kébir, L. Cheslow, M. Chabarati, C. Larochelle, and A. Prat, “JAML mediates monocyte and CD8 T cell migration across the brain endothelium,” *Ann. Clin. Transl. Neurol.*, 2015, doi: 10.1002/acn3.255.

[28] D. K. Newman, C. Hamilton, and P. J. Newman, “Inhibition of antigen-receptor signaling by Platelet Endothelial Cell Adhesion Molecule-1 (CD31) requires functional ITIMs, SHP-2, and p56lck,” *Blood*, vol. 97, no. 8, pp. 2351–2357, Apr. 2001, doi: 10.1182/blood.V97.8.2351.

[29] A. Mirshafiey, B. Asghari, G. Ghalamfarsa, F. J. Niaragh, and G. Azizi, “The Significance of Matrix Metalloproteinases in the Immunopathogenesis and Treatment of Multiple Sclerosis,” *Sultan Qaboos Univ. Med. J.*, vol. 14, no. 1, pp. 13–25, Feb. 2014, doi: 10.12816/0003332.

[30] S. McQuaid and J. Kirk, “The blood–brain barrier in multiple sclerosis,” *Int. Congr. Ser.*, vol. 1277, pp. 235–243, Apr. 2005, doi: 10.1016/j.ics.2005.02.026.

[31] J. R. Privratsky, D. K. Newman, and P. J. Newman, “PECAM-1: Conflicts of interest in inflammation,” *Life Sci.*, vol. 87, no. 3–4, pp. 69–82, Jul. 2010, doi: 10.1016/j.lfs.2010.06.001.

[32] T. G. Walsh *et al.*, “Stabilization of brain microvascular endothelial barrier function by shear stress involves VE-cadherin signaling leading to modulation of pTyr-occludin levels,” *J. Cell. Physiol.*, vol. 226, no. 11, pp. 3053–3063, Nov. 2011, doi: 10.1002/jcp.22655.

[33] M. Fujimoto *et al.*, “Tissue Inhibitor of Metalloproteinases Protect Blood—Brain Barrier Disruption in Focal Cerebral Ischemia,” *J. Cereb. Blood Flow Metab.*, vol. 28, no. 10, pp. 1674–1685, Oct. 2008, doi: 10.1038/jcbfm.2008.59.

[34] J. I. Alvarez, R. Cayrol, and A. Prat, “Disruption of central nervous system barriers in multiple sclerosis,” *Biochim. Biophys. Acta - Mol. Basis Dis.*, vol. 1812, no. 2, pp. 252–264, Feb. 2011, doi: 10.1016/j.bbadis.2010.06.017.

[35] S. M. Stamatovic, O. B. Dimitrijevic, R. F. Keep, and A. V. Andjelkovic, “Inflammation and brain edema: New insights into the role of chemokines and their receptors,” *Acta Neurochirurgica, Supplementum*. 2006, doi: 10.1007/3-211-30714-1_91.

[36] W. Abdullahi, D. Tripathi, and P. T. Ronaldson, “Blood-brain barrier dysfunction in ischemic stroke: targeting tight junctions and transporters for vascular protection,” *Am. J. Physiol. Physiol.*, vol. 315, no. 3, pp. C343–C356, Sep. 2018, doi: 10.1152/ajpcell.00095.2018.

[37] E. J. Mufson *et al.*, “Gene Profiling of Nucleus Basalis Tau Containing Neurons in Chronic Traumatic Encephalopathy: A Chronic Effects of Neurotrauma Consortium Study,” *J. Neurotrauma*, vol. 35, no. 11, pp. 1260–1271, Jun. 2018, doi: 10.1089/neu.2017.5368.

[38] T. Ikezu *et al.*, “Affinity-purification and characterization of caveolins from the brain: Differential expression of caveolin-1, -2, and -3 in brain endothelial and astroglial cell types,” *Brain Res.*, vol. 804, no. 2, pp. 177–192, Sep. 1998, doi: 10.1016/S0006-8993(98)00498-3.

[39] J. Badaut, D. O. Ajao, D. W. Sorensen, A. M. Fukuda, and L. Pellerin, “Caveolin expression changes in the neurovascular unit after juvenile traumatic brain injury: Signs of blood–brain barrier healing?,” *Neuroscience*, vol. 285, pp. 215–226, Jan. 2015, doi: 10.1016/j.neuroscience.2014.10.035.

[40] M. M. Hill *et al.*, “PTRF-Cavin, a Conserved Cytoplasmic Protein Required for Caveola Formation and Function,” *Cell*, 2008, doi: 10.1016/j.cell.2007.11.042.

[41] E. K. Bosma, C. J. F. van Noorden, R. O. Schlingemann, and I. Klaassen, “The role of plasmalemma vesicle-associated protein in pathological breakdown of blood–brain and blood–retinal barriers: potential novel therapeutic target for cerebral edema and diabetic macular edema,” *Fluids Barriers CNS*, vol. 15, no. 1, p. 24, Dec. 2018, doi: 10.1186/s12987-018-0109-2.

[42] Q. Zhu, M. Yamakuchi, and C. J. Lowenstein, “SNAP23 regulates endothelial exocytosis of von Willebrand Factor,” *PLoS One*, 2015, doi: 10.1371/journal.pone.0118737.

[43] S. A. Liddelow *et al.*, “Molecular Characterisation of Transport Mechanisms at the Developing Mouse Blood–CSF Interface: A Transcriptome Approach,” *PLoS One*, vol. 7, no. 3, p. e33554, Mar. 2012, doi: 10.1371/journal.pone.0033554.

[44] D. Feng, R. Flaumenhaft, C. Bandeira-Melo, P. F. Weller, and A. M. Dvorak, “Ultrastructural localization of vesicle-associated membrane protein(s) to specialized membrane structures in human pericytes, vascular smooth muscle cells endothelial cells, neutrophils, and eosinophils,” *J. Histochem. Cytochem.*, 2001, doi: 10.1177/002215540104900303.
