# Supplementary material for: Paracellular and Transcellular Leukocytes Diapedesis Are Divergent but Interconnected Evolutionary Events
Source: Genes (Basel). 2021 Feb 10;12(2):254. doi: 10.3390/genes12020254 (PMC7916592; doi:10.3390/genes12020254)
Supplement: Supplementary file 1 [file genes-12-00254-s001.zip › suplementary_3adera/supplemantary_3_3adera(SDP).zip!/SDPpred_pecam1_macrious.pdf]

SDPpred: Results

Results: List of SDPs

	Alignment position	Amino acid in PCEAM-1-HUMAN-MKR	Mutual information (I_p)	Z-score (Z_p)	
1	15	11Met	4.92e-01	2.15	Details
2	352	343Leu	5.33e-01	2.11	Details
3	649	619Ala	5.41e-01	2.09	Details
4	424	404Val	5.34e-01	2.07	Details
5	347	338Asp	5.86e-01	2.06	Details
6	253	246Ile	4.68e-01	2.06	Details
7	679	647Ser	5.44e-01	2.04	Details
8	669	637Met	5.58e-01	2.03	Details
9	131	125Val	5.09e-01	2.03	Details
10	668	636Glu	5.75e-01	2.02	Details
11	670	638Ser	5.51e-01	2.00	Details
12	657	627Lys	4.98e-01	1.99	Details
13	198	192Glu	5.40e-01	1.99	Details
14	373	357Phe	5.06e-01	1.96	Details
15	208	202Leu	4.86e-01	1.92	Details
16	205	199Asp	5.62e-01	1.88	Details
17	422	402Ile	4.99e-01	1.83	Details

18	510	483Ser	5.35e-01	1.83	Details
19	724	680Asn	5.21e-01	1.82	Details
20	442	420Glu	5.39e-01	1.82	Details
21	194	188Phe	5.66e-01	1.81	Details
22	200	194Pro	5.57e-01	1.81	Details
23	490	466Asp	5.51e-01	1.80	Details
24	725	681Lys	5.31e-01	1.80	Details
25	93	87Ser	5.18e-01	1.79	Details
26	147	141Glu	5.52e-01	1.79	Details
27	661	630Ala	5.40e-01	1.79	Details
28	76	70Gln	5.33e-01	1.78	Details
29	678	646Asn	5.20e-01	1.77	Details
30	367	355Ala	5.09e-01	1.77	Details
31	166	160Pro	5.55e-01	1.77	Details
32	210	204Phe	5.28e-01	1.76	Details
33	509	482His	5.69e-01	1.76	Details
34	782	718Lys	5.32e-01	1.76	Details
35	39	35Asn	5.16e-01	1.76	Details
36	346	337Leu	5.10e-01	1.74	Details
37	400	380Asp	5.32e-01	1.73	Details
38	53	49Asn	5.24e-01	1.72	Details
39	240	233Phe	5.76e-01	1.72	Details
40	355	346Ser	5.01e-01	1.71	Details
41	330	323Glu	5.19e-01	1.71	Details
42	146	140Lys	5.22e-01	1.70	Details
43	607	577Arg	5.37e-01	1.70	Details
44	278	271Ile	5.43e-01	1.70	Details
45	173	167Lys	5.27e-01	1.70	Details
46	737	683Pro	5.68e-01	1.68	Details

47	587	557Trp	5.66e-01	1.68	Details
48	765	701His	5.45e-01	1.67	Details
49	632	602Gly	5.55e-01	1.67	Details
50	234	228Thr	5.50e-01	1.67	Details
51	414	394Val	4.95e-01	1.67	Details
52	247	240Ile	4.81e-01	1.66	Details
53	326	319Val	4.64e-01	1.65	Details
54	671	639Arg	5.15e-01	1.65	Details
55	553	525Val	4.74e-01	1.63	Details
56	267	260Val	5.40e-01	1.62	Details
57	781	717Arg	5.32e-01	1.62	Details
58	319	312Ser	5.40e-01	1.58	Details
59	334	327Lys	5.10e-01	1.57	Details
60	145	139Lys	5.14e-01	1.56	Details
61	663	631Lys	4.92e-01	1.56	Details
62	774	710Glu	5.15e-01	1.55	Details
63	760	696Ser	5.06e-01	1.55	Details
64	313	306Val	5.21e-01	1.49	Details
65	482	459Asn	5.32e-01	1.48	Details
66	283	276Lys	5.49e-01	1.46	Details
67	410	390Ile	5.40e-01	1.31	Details
68	449	427Ile	5.31e-01	1.30	Details
69	156	150Val	5.29e-01	1.29	Details
70	642	612Ile	4.86e-01	1.26	Details
71	426	406Glu	5.49e-01	1.26	Details
72	399	379Ser	5.51e-01	1.24	Details
73	245	238Phe	5.15e-01	1.22	Details
74	412	392Lys	5.59e-01	1.22	Details
75	498	471Asp	5.74e-01	1.22	Details
76	761	697Ser	5.53e-01	1.21	Details

77	707	667Asp	5.68e-01	1.21	Details
78	49	45Trp	5.73e-01	1.21	Details
79	638	608Ile	5.56e-01	1.20	Details
80	114	108Lys	5.12e-01	1.20	Details
81	214	208Ala	5.61e-01	1.20	Details
82	603	573Thr	5.48e-01	1.20	Details
83	251	244Gly	5.19e-01	1.19	Details
84	677	645Leu	5.33e-01	1.18	Details
85	95	89Lys	5.35e-01	1.18	Details
86	401	381Ser	5.27e-01	1.18	Details
87	586	556Phe	5.56e-01	1.18	Details
88	530	503Val	5.64e-01	1.18	Details
89	567	539Glu	5.37e-01	1.18	Details
90	372	356Asn	5.31e-01	1.17	Details
91	425	405Cys	5.76e-01	1.16	Details
92	654	624Phe	5.76e-01	1.15	Details
93	500	473Glu	5.33e-01	1.15	Details
94	226	220Glu	5.44e-01	1.14	Details
95	203	197Glu	5.24e-01	1.13	Details
96	625	595Ile	5.58e-01	1.13	Details
97	646	616Leu	5.05e-01	1.12	Details
98	143	137Leu	4.98e-01	1.10	Details
99	640	610Gly	5.38e-01	1.10	Details
100	177	171Asn	5.42e-01	1.10	Details
101	238	232Ser	5.61e-01	1.09	Details
102	483	460Asp	5.15e-01	1.08	Details
103	136	130Val	4.93e-01	1.08	Details
104	543	515Ser	5.17e-01	1.06	Details
105	43	39Met	4.78e-01	1.05	Details

106	717	673Ala	5.06e-01	1.03	Details
107	445	423Lys	5.28e-01	1.02	Details
108	296	289Val	5.23e-01	1.01	Details
109	160	154Val	5.32e-01	0.96	Details
110	165	159Ala	5.46e-01	0.93	Details
111	780	716Val	4.88e-01	0.89	Details
112	676	644Leu	3.25e-01	0.88	Details
113	518	491Val	5.39e-01	0.85	Details
114	138	132Ser	5.11e-01	0.84	Details
115	153	147Ile	5.68e-01	0.76	Details
116	718	674Met	5.63e-01	0.75	Details
117	358	349Ile	5.38e-01	0.75	Details
118	258	251Gln	5.30e-01	0.74	Details
119	300	293Met	5.35e-01	0.72	Details
120	521	494Val	5.38e-01	0.70	Details
121	682	650Glu	2.54e-01	0.69	Details
122	303	296Val	5.35e-01	0.69	Details
123	360	351Gly	5.76e-01	0.67	Details
124	174	168Leu	5.72e-01	0.66	Details
125	231	225Glu	5.51e-01	0.65	Details
126	71	65Ser	5.43e-01	0.65	Details
127	696	658Glu	5.35e-01	0.65	Details
128	317	310Arg	5.32e-01	0.64	Details
129	201	195Val	5.34e-01	0.63	Details
130	129	123Tyr	5.52e-01	0.62	Details
131	302	295Met	5.45e-01	0.62	Details
132	13	9Ala	3.23e-01	0.61	Details
133	589	559Lys	5.55e-01	0.61	Details
134	374	358Thr	5.37e-01	0.61	Details
135	182	176Lys	5.32e-01	0.60	Details

136	576	547Gln	5.26e-01	0.60	Details
137	667	635Val	5.55e-01	0.59	Details
138	11	7Gln	3.25e-01	0.59	Details
139	687	655Pro	5.71e-01	0.59	Details
140	383	363Asp	5.39e-01	0.58	Details
141	566	538Arg	5.48e-01	0.58	Details
142	685	653Ser	2.54e-01	0.58	Details
143	260	253His	5.45e-01	0.58	Details
144	172	166Glu	5.42e-01	0.58	Details
145	287	280Ala	5.29e-01	0.57	Details
146	365	353Pro	5.41e-01	0.57	Details
147	569	541Glu	5.55e-01	0.57	Details
148	683	651Lys	2.55e-01	0.56	Details
149	10	6Ala	3.24e-01	0.56	Details
150	12	8Gly	3.28e-01	0.55	Details
151	615	585Pro	5.29e-01	0.55	Details
152	299	292Val	5.02e-01	0.54	Details
153	528	501Asp	5.38e-01	0.54	Details
154	290	283Arg	5.46e-01	0.54	Details
155	395	375Ile	5.34e-01	0.54	Details
156	98	92Glu	5.56e-01	0.53	Details
157	698	660Asn	5.19e-01	0.53	Details
158	27	23Ser	5.01e-01	0.53	Details
159	592	562Ala	5.14e-01	0.53	Details
160	597	567Glu	5.16e-01	0.53	Details
161	481	458Ser	5.34e-01	0.52	Details
162	157	151Asn	5.40e-01	0.52	Details
163	32	28Gln	5.28e-01	0.52	Details
164	388	368Gln	5.20e-01	0.52	Details

165	207	201Val	5.45e-01	0.52	Details
166	666	634Pro	5.19e-01	0.52	Details
167	211	205Arg	5.55e-01	0.52	Details
168	232	226Leu	5.65e-01	0.52	Details
169	715	671Asn	5.30e-01	0.52	Details
170	502	475Gln	5.45e-01	0.51	Details
171	484	461Pro	5.65e-01	0.51	Details
172	628	598Pro	5.48e-01	0.51	Details
173	175	169Glu	5.39e-01	0.51	Details
174	97	91Thr	5.34e-01	0.50	Details
175	390	370Gln	5.54e-01	0.50	Details
176	471	449Lys	5.29e-01	0.50	Details
177	99	93Ser	5.34e-01	0.50	Details
178	785	721Pro	5.37e-01	0.50	Details
179	33	29Glu	5.16e-01	0.50	Details
180	286	279Val	2.59e-01	0.49	Details
181	218	212Ser	5.24e-01	0.49	Details
182	508	481Cys	5.65e-01	0.49	Details
183	24	20Leu	5.39e-01	0.48	Details
184	224	218Thr	5.49e-01	0.48	Details
185	126	120Thr	2.61e-01	0.47	Details
186	315	308Ser	5.29e-01	0.47	Details
187	517	490Glu	4.93e-01	0.47	Details
188	407	387Thr	5.44e-01	0.47	Details
189	269	262His	5.26e-01	0.46	Details
190	280	273Gln	5.20e-01	0.46	Details
191	67	63Thr	5.31e-01	0.46	Details
192	496	469Thr	5.09e-01	0.46	Details
193	621	591Thr	5.02e-01	0.46	Details
194	655	625Leu	5.47e-01	0.46	Details

195	409	389Gly	5.50e-01	0.45	Details
196	310	303Thr	5.10e-01	0.45	Details
197	580	551Asn	5.41e-01	0.45	Details
198	26	22Cys	5.51e-01	0.45	Details
199	161	155Pro	5.29e-01	0.44	Details
200	230	224Ser	5.11e-01	0.44	Details
201	246	239His	5.30e-01	0.44	Details
202	295	288Ala	5.26e-01	0.44	Details
203	168	162His	5.51e-01	0.44	Details
204	142	136Thr	5.13e-01	0.44	Details
205	506	479Asp	5.23e-01	0.43	Details
206	328	321Ile	2.43e-01	0.43	Details
207	244	237Lys	5.07e-01	0.43	Details
208	623	593Arg	5.34e-01	0.43	Details
209	44	40Lys	5.48e-01	0.43	Details
210	540	512Val	5.30e-01	0.43	Details
211	635	605Ala	5.10e-01	0.43	Details
212	279	272Ile	2.54e-01	0.43	Details
213	204	198Gln	5.33e-01	0.43	Details
214	525	498Ala	2.69e-01	0.43	Details
215	34	30Asn	5.29e-01	0.42	Details
216	533	506Ser	5.08e-01	0.42	Details
217	581	552Ala	5.04e-01	0.42	Details
218	164	158Lys	5.20e-01	0.42	Details
219	585	555Ala	5.05e-01	0.41	Details
220	348	339Gln	2.63e-01	0.41	Details
221	584	554Gln	4.99e-01	0.41	Details
222	70	64Thr	2.61e-01	0.41	Details
223	658	628Ala	5.41e-01	0.41	Details

224	788	724Val	5.61e-01	0.41	Details
225	121	115Asn	5.23e-01	0.41	Details
226	622	592Val	2.42e-01	0.41	Details
227	722	678Asn	2.68e-01	0.41	Details
228	284	277Ala	2.65e-01	0.40	Details
229	171	165Ile	2.72e-01	0.40	Details
230	652	622Cys	5.70e-01	0.40	Details
231	651	621Lys	5.42e-01	0.40	Details
232	90	84Asn	5.17e-01	0.40	Details
233	20	16Leu	5.19e-01	0.40	Details
234	343	334Phe	5.63e-01	0.39	Details
235	327	320Asn	5.10e-01	0.39	Details
236	536	509Ser	2.75e-01	0.39	Details
237	428	408Leu	2.57e-01	0.39	Details
238	220	214Ile	5.63e-01	0.38	Details
239	421	401Gln	5.52e-01	0.38	Details
240	583	553Thr	4.99e-01	0.37	Details
241	699	661Ser	5.42e-01	0.37	Details
242	55	51Lys	5.22e-01	0.37	Details
243	274	267Phe	5.40e-01	0.36	Details
244	167	161Ile	2.54e-01	0.35	Details
245	389	369Thr	5.32e-01	0.35	Details
246	403	383Thr	5.22e-01	0.35	Details
247	184	178Lys	5.36e-01	0.35	Details
248	594	564Lys	2.55e-01	0.35	Details
249	783	719Ala	5.09e-01	0.34	Details
250	227	221Ser	5.30e-01	0.33	Details
251	538	510Ser	5.39e-01	0.33	Details
252	123	117Glu	5.29e-01	0.33	Details
253	391	371Asp	5.54e-01	0.32	Details

254	213	207Gln	5.19e-01	0.32	Details
255	35	31Ser	4.97e-01	0.31	Details
256	185	179Arg	5.30e-01	0.30	Details
257	80	74Leu	4.92e-01	0.30	Details
258	48	44Asp	5.33e-01	0.30	Details
259	665	633Met	5.33e-01	0.30	Details
260	432	412Arg	5.27e-01	0.29	Details
261	42	38Asp	5.27e-01	0.29	Details
262	641	611Val	4.79e-01	0.29	Details
263	195	189Val	4.89e-01	0.28	Details
264	491	467Asn	5.27e-01	0.25	Details
265	689	657Met	4.91e-01	0.22	Details
266	275	268Pro	5.24e-01	0.21	Details
267	366	354Pro	5.20e-01	0.21	Details
268	324	317Ile	4.95e-01	0.21	Details
269	473	451Leu	5.09e-01	0.20	Details
270	572	544Pro	3.57e-01	0.17	Details
271	119	113Val	4.59e-01	0.16	Details
272	614	584Val	4.91e-01	0.14	Details
273	753	690Tyr	1.94e-01	0.12	Details
274	193	187Asn	1.96e-01	0.12	Details
275	223	217Gln	3.45e-01	0.10	Details
276	555	527Glu	1.84e-01	0.10	Details
277	304	297Glu	1.92e-01	0.10	Details
278	252	245Met	3.49e-01	0.09	Details
279	181	175Val	3.51e-01	0.09	Details
280	276	269Glu	1.92e-01	0.08	Details
281	393	373Thr	3.40e-01	0.08	Details
282	700	662His	3.64e-01	0.07	Details

283	128	122Glu	3.43e-01	0.07	Details
284	601	571Tyr	1.94e-01	0.07	Details
285	152	146Gly	3.54e-01	0.07	Details
286	338	331Glu	3.36e-01	0.07	Details
287	721	677Ile	3.58e-01	0.06	Details
288	37	33Thr	1.87e-01	0.06	Details
289	36	32Phe	1.85e-01	0.06	Details
290	241	234Ser	3.43e-01	0.06	Details
291	763	699Glu	3.47e-01	0.06	Details
292	719	675Lys	1.90e-01	0.06	Details
293	103	97Pro	1.91e-01	0.06	Details
294	656	626Arg	3.38e-01	0.06	Details
295	773	709Thr	1.78e-01	0.06	Details
296	85	79Asp	3.65e-01	0.06	Details
297	779	715Glu	1.81e-01	0.06	Details
298	446	424Gly	1.99e-01	0.06	Details
299	312	305Lys	1.97e-01	0.05	Details
300	703	663Tyr	3.60e-01	0.05	Details
301	45	41Ser	3.41e-01	0.05	Details
302	100	94Tyr	2.03e-01	0.05	Details
303	697	659Ala	3.36e-01	0.05	Details
304	285	278Ile	1.88e-01	0.05	Details
305	314	307Glu	1.86e-01	0.04	Details
306	532	505Ile	3.31e-01	0.04	Details
307	637	607Val	3.17e-01	0.04	Details
308	162	156Glu	2.00e-01	0.04	Details
309	77	71His	1.85e-01	0.04	Details
310	758	695Val	2.02e-01	0.04	Details
311	650	620Ala	3.28e-01	0.04	Details
312	84	78Asp	1.82e-01	0.04	Details

313	141	135Val	3.25e-01	0.03	Details
314	83	77Lys	1.95e-01	0.03	Details
315	776	712Val	1.96e-01	0.03	Details
316	329	322Thr	1.88e-01	0.03	Details
317	58	54Thr	3.34e-01	0.03	Details
318	634	604Ile	1.88e-01	0.03	Details
319	17	13Leu	1.90e-01	0.03	Details
320	46	42Leu	3.57e-01	0.03	Details
321	420	400Val	2.02e-01	0.02	Details
322	354	345Leu	1.67e-01	0.02	Details
323	620	590Leu	3.31e-01	0.02	Details
324	376	360Gln	3.55e-01	0.02	Details
325	520	493Arg	3.45e-01	0.02	Details
326	775	711Thr	1.88e-01	0.01	Details
327	199	193Phe	1.75e-01	0.01	Details
328	778	714Ser	1.89e-01	0.01	Details
329	747	688Val	2.01e-01	0.01	Details
330	357	348Ser	1.95e-01	0.01	Details
331	301	294Ala	1.81e-01	0.01	Details
332	616	586Arg	3.28e-01	0.01	Details
333	768	704Leu	3.58e-01	0.01	Details
334	47	43Pro	5.74e-03	0.00	Details
335	51	47Val	4.15e-03	0.00	Details
336	54	50Gly	6.66e-03	0.00	Details
337	61	57Cys	6.25e-03	0.00	Details
338	66	62Ser	4.35e-03	0.00	Details
339	106	100Arg	4.68e-03	0.00	Details
340	111	105Gly	6.66e-03	0.00	Details
341	113	107Tyr	7.82e-03	0.00	Details

342	115	109Cys	6.25e-03	0.00	Details
343	117	111Val	4.15e-03	0.00	Details
344	124	118Lys	4.17e-03	0.00	Details
345	135	129Gly	6.66e-03	0.00	Details
346	139	133Pro	5.74e-03	0.00	Details
347	151	145Gly	6.66e-03	0.00	Details
348	158	152Cys	6.25e-03	0.00	Details
349	163	157Glu	6.37e-03	0.00	Details
350	169	163Phe	4.31e-03	0.00	Details
351	212	206Cys	6.25e-03	0.00	Details
352	225	219Ser	4.35e-03	0.00	Details
353	243	236Pro	5.74e-03	0.00	Details
354	249	242Pro	5.74e-03	0.00	Details
355	255	248Glu	6.37e-03	0.00	Details
356	256	249Gly	6.66e-03	0.00	Details
357	263	256Cys	6.25e-03	0.00	Details
358	297	290Tyr	7.82e-03	0.00	Details
359	306	299Ser	4.35e-03	0.00	Details
360	307	300Gly	6.66e-03	0.00	Details
361	309	302Tyr	7.82e-03	0.00	Details
362	311	304Cys	6.25e-03	0.00	Details
363	320	313Lys	4.17e-03	0.00	Details
364	331	324Leu	4.27e-03	0.00	Details
365	332	325Phe	4.31e-03	0.00	Details
366	335	328Pro	5.74e-03	0.00	Details
367	337	330Leu	4.27e-03	0.00	Details
368	356	347Cys	6.25e-03	0.00	Details
369	396	376Ala	3.75e-03	0.00	Details
370	402	382Gly	6.66e-03	0.00	Details
371	404	384Tyr	7.82e-03	0.00	Details

372	406	386Cys	6.25e-03	0.00	Details
373	408	388Ala	3.75e-03	0.00	Details
374	415	395Lys	4.17e-03	0.00	Details
375	417	397Ser	4.35e-03	0.00	Details
376	429	409Ser	4.35e-03	0.00	Details
377	431	411Pro	5.74e-03	0.00	Details
378	453	431Cys	6.25e-03	0.00	Details
379	458	436Gly	6.66e-03	0.00	Details
380	461	439Pro	5.74e-03	0.00	Details
381	462	440Ile	4.18e-03	0.00	Details
382	485	462Ala	3.75e-03	0.00	Details
383	487	464Phe	4.31e-03	0.00	Details
384	503	476Cys	6.25e-03	0.00	Details
385	505	478Ala	3.75e-03	0.00	Details
386	507	480Asn	3.90e-03	0.00	Details
387	516	489Ser	4.35e-03	0.00	Details
388	519	492Leu	4.27e-03	0.00	Details
389	523	496Val	4.15e-03	0.00	Details
390	524	497Ile	4.18e-03	0.00	Details
391	526	499Pro	5.74e-03	0.00	Details
392	544	516Gly	6.66e-03	0.00	Details
393	551	523Cys	6.25e-03	0.00	Details
394	556	528Gly	6.66e-03	0.00	Details
395	559	531Pro	5.74e-03	0.00	Details
396	560	532Ile	4.18e-03	0.00	Details
397	598	568Gly	6.66e-03	0.00	Details
398	600	570Tyr	7.82e-03	0.00	Details
399	602	572Cys	6.25e-03	0.00	Details
400	604	574Ala	3.75e-03	0.00	Details

401	606	576Asn	3.90e-03	0.00	Details
402	608	578Ala	3.75e-03	0.00	Details
403	617	587Ser	4.35e-03	0.00	Details
404	624	594Val	4.15e-03	0.00	Details
405	626	596Leu	4.27e-03	0.00	Details
406	627	597Ala	3.75e-03	0.00	Details
407	629	599Trp	7.43e-03	0.00	Details
408	631	601Lys	4.17e-03	0.00	Details
409	704	664Gly	6.66e-03	0.00	Details
410	754	691Thr	4.82e-03	0.00	Details
411	755	692Glu	6.37e-03	0.00	Details
412	756	693Val	4.15e-03	0.00	Details
413	777	713Tyr	7.82e-03	0.00	Details
414	298	291Ser	1.84e-01	-0.00	Details

If you use these results, please cite:
Kalinina OV, Mironov AA, Gelfand MS, Rakhmaninova AB. (2004) Automated selection of positions determining functional specificity of proteins by comparative analysis of orthologous groups in protein families. *Protein Sci* **13(2):** 443-56.

Mail to: sdppred@belozersky.msu.ru

back to home page
